# The design of Mn^2+^&Co^2+^ co-doped CdTe quantum dot sensitized solar cells with much higher efficiency†

**DOI:** 10.1039/d0ra06381a

**Published:** 2020-09-29

**Authors:** Huazheng Li, Wangwei Lu, Bin Song, Jing Zhou, Gaoling Zhao, Gaorong Han

**Affiliations:** State Key Laboratory of Silicon Materials & School of Materials Science and Engineering, Zhejiang University Hangzhou 310027 P. R. China glzhao@zju.edu.cn; State Key Laboratory of Silicon Materials & Department of Physics, Zhejiang University Hangzhou 310027 P. R. China; Department of Traffic Management Engineering, Zhejiang Police College Hangzhou 310053 P. R. China

## Abstract

High quality Mn^2+^-doped CdTe quantum dots (QDs), Co^2+^-doped CdTe QDs and Mn^2+^&Co^2+^ co-doped CdTe QDs were successfully synthesized *via* an aqueous phase method with mercaptopropanoic acid (MPA) ligands. The doped QDs maintain the same zinc blende structure of CdTe by X-ray diffraction (XRD). The Mn^2+^-doped CdTe QDs and Co^2+^-doped CdTe QDs both show a red-shift on absorption and photoluminescence (PL) spectra compared to pure CdTe QDs. In addition, Mn^2+^-doped CdTe QDs show a significant increase in the PL lifetime due to an orbitally forbidden d–d transition, which is of benefit to the reduction of electron recombination loss. Co^2+^ doping has a more matched doping energy level. In view of this, Mn^2+^&Co^2+^ co-doped CdTe QDs were applied as sensitizers for quantum dot sensitized solar cells, resulting in a significantly enhanced efficiency.

## Abstract 2

Mn^2+^&Co^2+^ co-doped CdTe QDs were successfully synthesized *via* aqueous phase method with mercaptopropanoic acid, which could generate different doping energy levels, and as sensitizers applied for QDSCs, showing a significantly enhanced efficiency.

## Graphical abstract

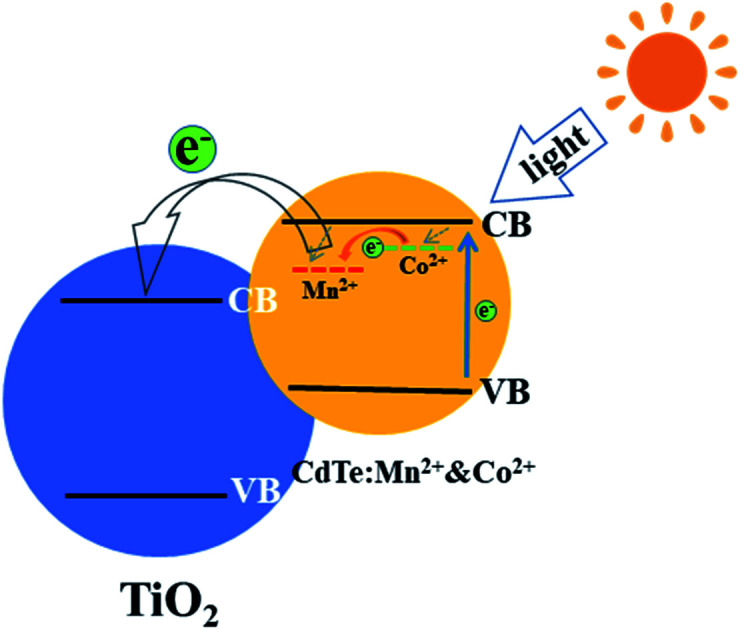

## 1. Introduction

Quantum dots (QDs) have exhibited properties significantly different from their bulk materials.^1–4^ The importance of these QDs is attributed to their unique properties such as band-gap tenability^2–4^ and multiexciton generation (MEG).^5–7^ Quantum dot sensitized solar cells (QDSCs)^8–12^ have attracted much attention because of their promising development prospects in solar cells. Zn–Cu–In–Se QDSCs retain a power conversion efficiency (PCE) record of 12.98%.^13^ On the other hand, CdTe QDs have advantages of high extinction coefficient (∼10^5^ cm^−1^)^14^ and convenient synthesis.^15^ In addition, CdTe has a narrower band gap (1.5 eV),^16^ which expands the light absorption range to a longer wavelength, and has a higher conduction band edge makes the electrons injection from CdTe QDs to TiO_2_ photoanode faster.^17^ CdTe QDs is a promising sensitizer in QDSCs. So far, however, only a few research studies on CdTe based on QDSCs have been carried out.^7,17^ The potential of CdTe based QDSCs has not been fully realized. Nowadays, introduction of dopant in semiconductors is widely used to modify the properties of host semiconductor nanocrystals such as electrical conductivity and magnetic properties.^18–22^ Doping of 3d transition metal in II–VI semiconductor QDs have been widely investigated because the dopant can adjust optical properties of QDs.^23–25^ The optical properties of transition metal doped QDs are found a dramatical change due to the presence of d-emission that arises by d–d transition.^26^

The transition between ^4^T_1_–^6^A_1_ of Mn^2+^ is both spin and orbitally forbidden, as a result, the emission lifetime of Mn^2+^-doped QDs measured to be in the range of milliseconds.^27^ Long excited state lifetime is one of the key factors for the design and development of higher efficient QDSCs. In addition, the doping of transition metals could accelerate the electron transfer speed and reduce the electron recombination rate at the TiO_2_/electrolyte interface. Mn^2+^ doped QDs have applied in QDSCs with good performance.^27–29^ On the other hand, doping a certain amount of cobalt could change energy level structure of semiconductor,^30^ adjust the Fermi level of photoanode,^31^ enhance light harvesting range,^32^ and improve the performance of DSSCs.

Considering the synergy of transition metals,^24,33^ the different doping energy levels make it easier for electrons to transition. In this paper, Co^2+^ and Mn^2+^ were chosen as effective dopants for enhancing the performance of QDSCs. Doped QDs through adding dopant precursor during the syntheses. For the Mn^2+^&Co^2+^ co-doped CdTe QDs sensitized TiO_2_ photoelectrodes in polysulfide redox electrolyte, cell performance with 2.26% efficiency was obtained. A systematic study was also undertaken on the effects of doping on the recombination mechanism by optical absorption spectra, photoluminescence (PL) and PL decay spectra.

## 2. Experiment

### 2.1. Synthesis of doped CdTe QDs

CdTe QDs and doped CdTe QDs were synthesized by hot injection method. In a typical Te precursor preparation procedure, Te powder (4 mmol) and sodium borohydride (10 mmol) were dissolved in 2.5 ml distilled water at 30 °C under nitrogen atmosphere for 3 h. To synthesize pure CdTe QDs, Cd(NO_3_)_2_ (4 mmol) was dissolved in 80 ml distilled water with mercaptopropionic acid (MPA, 8 mmol), drop into NaOH solution (1 M) to adjust the pH to 6. Then stirred 30 min at 80 °C in a 250 ml three-neck round-bottom flask under nitrogen atmosphere, and injected Te precursor rapidly, after reacting at 100 °C for 24 hours. The reaction mixture was then purified by centrifugation at 6000 rpm for 5 min with adding isovolumetric 2-propanol. Take the supernatant and centrifugate at 8000 rpm for 5 min to obtain precipitation of the MPA capped pure CdTe QDs, then disperse with distilled water, labels CdTe-pure, to obtain Mn^2+^, Co^2+^ doped or co-doped in CdTe QDs, MnCl_2_, CoCl_2_ or both with Cd(NO_3_)_2_ and MPA dissolved in distilled water [*n*(Mn) : *n*(Cd) = 1 : 19, *n*(Co) : *n*(Cd) = 1 : 19, *n*(Mn) : *n*(Co) : *n*(Cd) = 1 : 1 : 18 and total cations remain 4 mmol], kept next synthesize steps same, and label CdTe:Mn^2+^, CdTe:Co^2+^ and CdTe:Mn^2+^&Co^2+^, respectively. All QDs were stored in distilled water.

### 2.2. Construction of QDSCs

TiO_2_ mesoporous films were prepared by screen printing of a transparent layer (12 ± 0.5 μm) with the use of homemade P25 paste over F:SnO_2_ glass (FTO, 10 Ω per square) substrates followed by sintered in a muffle-type furnace at 500 °C for 30 min. The obtained TiO_2_ films were then sensitized using QDs. The TiO_2_ films were dipped into pure CdTe QDs or different doped QDs aqueous solution and remained for 24 h to tether QDs on TiO_2_ films before being rinsed with water and ethanol sequentially.

The cells were prepared by assembling the counter electrode and a QD-sensitized photoanode together with polysulfide electrolyte between them. The Cu_2_S counter electrodes were obtained by immersing brass foil in HCl solution (1.0 M) at 70 °C for 5 min, which were then vulcanized by insertion into a polysulfide electrolyte solution for 10 min. The composition of the polysulfide electrolyte solution was 1.0 M Na_2_S and 1.0 M S in distilled water.

### 2.3. Characterization

Powder X-ray diffraction (XRD) was employed to characterize phase and crystallinity of the samples. Data were collected on an X'Pert PRO X-ray diffractometer with Cu Kα radiation (*λ* = 1.54178 Å) at a beam current of 40 mA. DI X-ray photoelectron spectroscopy (XPS) was used to analyze the composition of the samples. High resolution transmission electronic microscopy (HRTEM) carried out on Tecnai F20 was used to observe the morphology and microstructure of the samples. In order to clarify the doping concentration in CdTe QDs, the actual Mn^2+^ and Co^2+^ concentration was measured for the samples by Inductively Coupled Plasma (ICP) Atomic Emission Spectroscopy (730-ES). The optical transmittance spectra of the samples were recorded on a Cary 5000 UV-Vis-NIR spectrophotometer. Photoluminescence (PL) spectra and photoluminescence decay measurement were achieved by an FLS920 fluorescence emission spectrophotometer. The photovoltaic performance of the QDSCs were evaluated using a Keithley 2400 source meter with illumination by a 150 W AM 1.5G solar simulator. The power of the simulated solar light was calibrated to 100 mW cm^−2^ with the use of an optical power meter. Multiple devices were fabricated and all had similar performance, and took the average. The photoactive area was 0.160 cm^2^.

## 3. Results and discussions

### 3.1. Microstructure of Mn^2+^ and Co^2+^ doped CdTe quantum dots

**Fig. 1:**
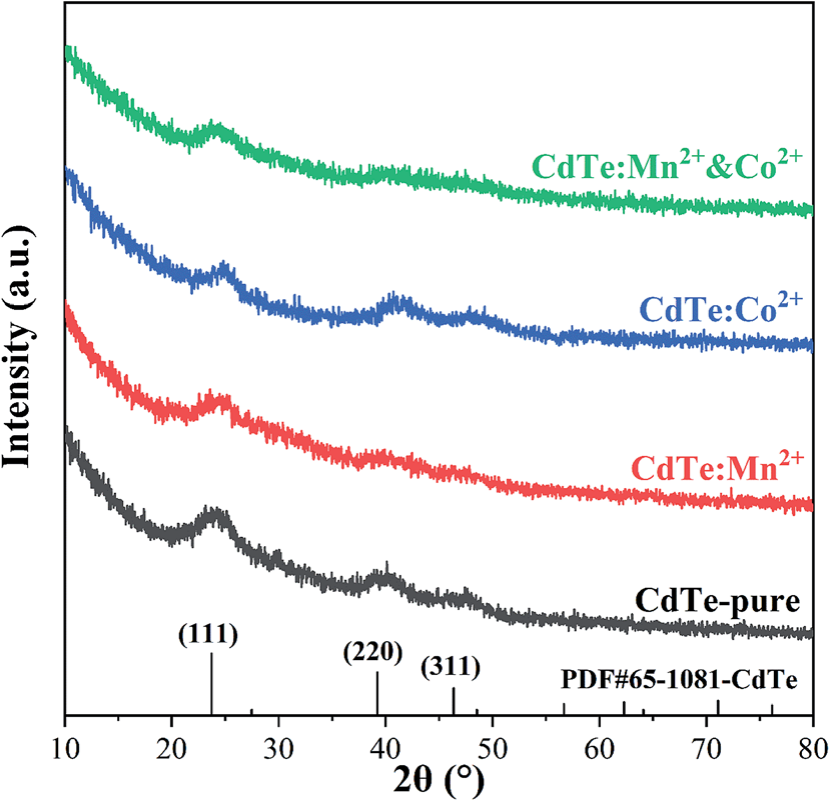
XRD patterns of pure CdTe QDs (CdTe-pure) and various doped QDs (CdTe:Mn^2+^, CdTe:Co^2+^ and CdTe:Mn^2+^&Co^2+^).

Various amounts of doping precursor were added in the starting solution to investigate the effects of doping concentration on properties. The results showed that 5 mol% doping concentration in the precursor got the best performance. Therefore, 5 mol% is chosen as the doping concentration in the precursor in present work. Fig. 1 shows XRD patterns of pure CdTe QDs and various doped CdTe QDs (CdTe:Mn^2+^, CdTe:Co^2+^ and CdTe:Mn^2+^&Co^2+^). Three well-defined peaks were observed for all samples in Fig. 1 at 2*θ* = 24.7°, 41.6° and 49.3° correspond to (111), (220) and (311) planes of the cubic of bulk CdTe zinc blende phase (PDF# 65-1081), respectively. No extra peaks of impurity phases are observed for the doped samples, which indicates that doping does not introduce new crystal phase. However, comparing with those of pure CdTe, the diffraction peaks of the doped sample broaden, indicating that doping weaken the crystal intensity of CdTe QDs.

**Fig. 2:**
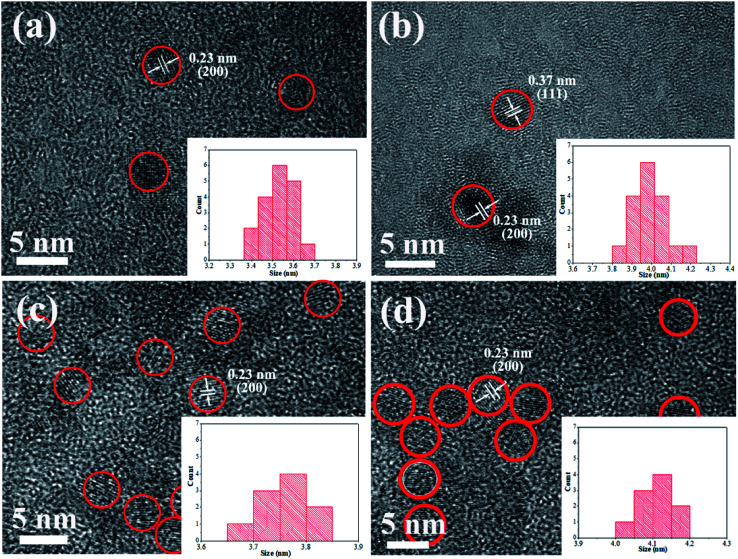
HRTEM images of pure CdTe QDs (a); the doped QDs prepared with Mn precursor (CdTe:Mn^2+^) (b); Co precursor (CdTe:Co^2+^) (c); and both Mn and Co precursor (CdTe:Mn^2+^&Co^2+^) (d). Insets: size distributions.

Fig. 2 shows HRTEM images of pure CdTe QDs and various doped CdTe QDs (CdTe:Mn^2+^, CdTe:Co^2+^ and CdTe:Mn^2+^&Co^2+^). Crystalline with distinct lattice distances are observed for all samples, exhibits (111) and (220) planes of CdTe zinc blende, which is consistent with XRD pattern (see Fig. 1), indicating that all samples have high crystallinity, and doping does not change the structure of CdTe QDs. Insets of Fig. 2 are size distribution of QDs. The results show that CdTe-pure, CdTe:Mn^2+^, CdTe:Co^2+^ and CdTe:Mn^2+^&Co^2+^ QDs own diameters ranged from 3.4 nm to 3.7 nm, 3.8 nm to 4.2 nm, 3.6 nm to 3.8 nm, and 4.0 nm to 4.2 nm, respectively. Doping enlarges the size of CdTe QD. The QDs size increases in the order as: CdTe-pure, CdTe:Co^2+^, CdTe:Mn^2+^, CdTe:Mn^2+^&Co^2+^. In addition, sizes of the particles are all less than the Bohr exciton radius of CdTe (∼7 nm (ref. 34)).

**Fig. 3:**
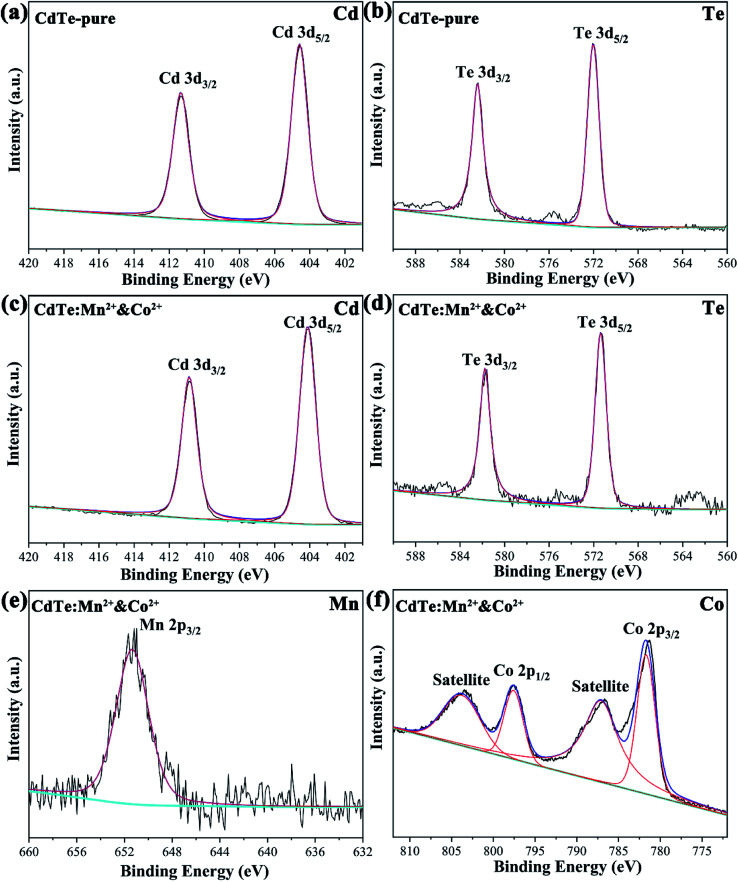
XPS spectra of the undoped CdTe QDs (CdTe-pure) (a and b) and the doped CdTe QDs (CdTe:Mn^2+^&Co^2+^) (c–f) binding energy spectra of Cd 3d (a and c), Te 3d (b and d), Mn 2p (e) and Co 2p (f).

Fig. 3 shows the typical XPS spectra of the undoped CdTe QDs (CdTe-pure) and the co-doped CdTe QDs (CdTe:Mn^2+^&Co^2+^). In Fig. 3a, the features at 404.9 and 411.6 eV for the undoped CdTe QDs are known to stem from Cd 3d_5/2_ and 3d_3/2_,^14,16^ respectively. The features at 571.9 and 582.4 eV of undoped CdTe QDs in Fig. 3b, corresponding to Te 3d_5/2_ and 3d_3/2_,^14,16^ respectively. Compared with undoped CdTe QDs, the binding energy of Cd and Te species in Fig. 3c and d for the co-doped QDs shifted to lower positions (Cd 3d shift form 404.9 and 411.6 eV to 404.1 and 410.9 eV, Te 3d shift form 571.9 and 582.4 eV to 571.4 eV and 581.7 eV). Fig. 3e and f are the high-resolution binding energy spectra for Mn and Co species, respectively. The peak of 652.2 eV, which can be attributed to Mn 2p, indicate the presence of Mn^2+^ species.^29,35^ The peaks observed at 795.1 eV and 782.3 eV can be assigned to Co 2p,^36^ and the satellite peaks (803.9 eV and 786.9 eV) also confirms the presence of Co^2+^ state. These results confirm the existence of Cd, Te, Mn and Co species in the co-doped QDs, and also indicate Mn^2+^ and Co^2+^ successfully doped in CdTe QDs. Moreover, based on ICP data, the actual cation ratio of Mn^2+^ and Cd^2+^ in the CdTe:Mn^2+^ is calculated to be 1.7 mol%, 98.3 mol%. The ratio of Co^2+^ and Cd^2+^ in the CdTe:Co^2+^ is calculated to be 2.2 mol%, 97.8 mol%, indicating that the actual doping concentration of Co^2+^ and Mn^2+^ is similar of a single doping. In co-doped QDs of CdTe:Mn^2+^&Co^2+^, the actual cation ratio of Mn^2+^, Co^2+^ and Cd^2+^ is calculated to be 3.0 mol%, 2.1 mol% and 94.9 mol%, respectively, also exhibiting that a similar doping concentration. The radii of Mn^2+^, Co^2+^, and Cd^2+^ ions are 0.67 nm, 0.745 nm, and 0.95 nm, respectively. A smaller radius gradient due to the presence of Co^2+^ as co-doping, which may causing Mn^2+^ is easier to dope into CdTe QDs.

### 3.2. Optical properties of Mn^2+^ and Co^2+^ doped CdTe quantum dots

**Fig. 4:**
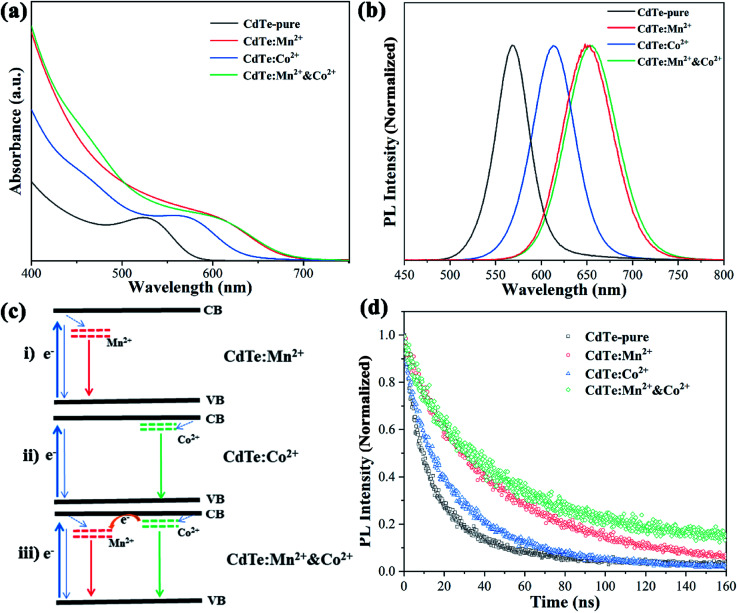
UV-vis absorption (a), photoluminescence (b) and photoluminescence decay spectra (d) of pure CdTe QDs and various doped QDs (CdTe:Mn^2+^, CdTe:Co^2+^ and CdTe:Mn^2+^&Co^2+^). Energy level diagram of the doped CdTe QDs (c).

Fig. 4a shows optical absorption spectra of pure CdTe QDs and various doped CdTe QDs (CdTe:Mn^2+^, CdTe:Co^2+^ and CdTe:Mn^2+^&Co^2+^). Compared to the pure CdTe QDs, the optical absorption in the visible range of the doped samples increases and the absorption edge of all doped samples shift towards longer wavelength. In addition, the samples exhibit absorption peaks at 527 nm, 571 nm, 609 nm, and 609 nm of CdTe-pure, CdTe:Co^2+^, CdTe:Mn^2+^ and CdTe:Mn^2+^&Co^2+^, respectively. The red shift of optical absorption can be ascribed to the size evolution with doping. As described in Section 3.1, the QDs size increases in the order as: CdTe-pure, CdTe:Co^2+^, CdTe:Mn^2+^, CdTe:Mn^2+^&Co^2+^. Smaller size QD own larger bandgap because of quantum size effect. Therefore the optical absorption red shift in the order as: CdTe-pure, CdTe:Co^2+^, CdTe:Mn^2+^, CdTe:Mn^2+^&Co^2+^. Fig. 4b shows photoluminescence (PL) spectra of various CdTe QDs excited at 405 nm. The pure CdTe QDs shows a narrow emission peak centered at 568 nm. The full width at half maximum (FWHM) is about 45 nm, indicating a narrow size distribution. The emission peaks of all doped QDs shift towards higher wavelength compared to pure CdTe QDs. The samples of CdTe:Co^2+^, CdTe:Mn^2+^ and CdTe:Mn^2+^&Co^2+^ exhibit the PL peaks at 613 nm, 651 nm and 654 nm, respectively.

Following electron transfer process can be suggested for the emission origin. When light irradiates, electrons absorbs the energy of the photons, and excited from the valence band (VB) to the conduction band (CB) of CdTe. The excited electrons on the CB of CdTe will relax to the doping energy level, then recombine with holes, and emit fluorescence. PL spectra (Fig. 4b) shows that the emission wavelength becomes longer in the order of CdTe:Mn^2+^ > CdTe:Co^2+^ > CdTe-pure, indicating that the doping energy levels below the CB of CdTe, and the doping energy level of Mn^2+^ lower than that of Co^2+^. Accordingly, the energy level diagram of Mn^2+^ and Co^2+^ doped CdTe QDs is given in Fig. 4c. When Mn^2+^ and Co^2+^ co-doped, two doping levels are existing simultaneously as shown in Fig. 4c(iii).

The photoluminescence quantum yields (PLQY) of samples were calculated based on PL data and optical absorbance data by using the internal standard method and rhodamine B as a standard reference. The results showed that CdTe-pure, CdTe:Mn^2+^, CdTe:Co^2+^ and CdTe:Mn^2+^&Co^2+^ owned PLQYs of 20.3%, 34.1%, 14.7% and 32.2%, respectively. The doping energy levels in the band gap will increase the carrier concentration, resulting in increased PLQY. However, electrons and holes are more likely to recombine at the doping energy level of Co^2+^, resulting in a decrease in PLQY.

Fig. 4d shows PL decay spectra of pure CdTe QDs and various doped QDs (CdTe:Mn^2+^, CdTe:Co^2+^ and CdTe:Mn^2+^&Co^2+^). The recovery times of excited states for all samples are in the range of nanoseconds. For decay to the same PL intensity, the all doped samples need more time as shown in Table 1, indicating that the lifetime of excited electrons relaxed to the doping level is longer than that at the conduction band,^24^ resulting in the emission lifetimes of the doped QDs are much longer than that of pure CdTe QDs, the measured values were fitted to the eqn (1) and the average lifetimes were calculated using eqn (2):1
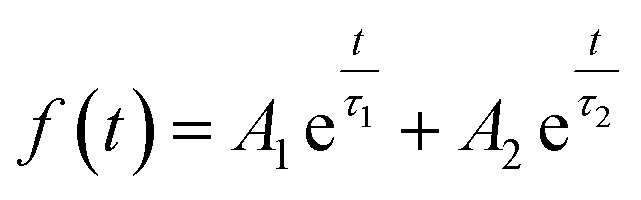

2 *τ*_avg_ = (*A*_1_*τ*_1_^2^ + *A*_2_*τ*_2_^2^)/(*A*_1_*τ*_1_ + *A*_2_*τ*_2_)

where *τ*_1_ and *τ*_2_ are the lifetimes, *A*_1_ and *A*_2_ are the contributions from the two components. The slow decay processes result from a sublevel of energy states below the conduction states of QDs caused by doped ions. In addition, due to the transition between ^4^T_1_–^6^A_1_ of Mn^2+^ is both spin and orbit forbidden,^27^ CdTe:Mn^2+^ shows a long excited state lifetime. When Mn^2+^ and Co^2+^ co-doped, the electrons located at the Co^2+^-doping energy level transit to the Mn^2+^-doping energy level, the samples can also exhibit a long emission lifetime. Compared with CdTe-pure, CdTe:Mn^2+^&Co^2+^ and CdTe:Mn^2+^ reduce the recombination of electrons and holes. Moreover, for samples with various doping amounts, Fig. S1† gives their optical absorption spectra and PL decay spectra, the results show that 5 mol% doping concentration in the Mn^2+^ precursor got the best performance.

**Table 1:** Fitted lifetimes of the CdTe-pure, CdTe:Mn^2+^, CdTe:Co^2+^ and CdTe:Mn^2+^&Co^2+^ samples

Samples	*A*_1_	*τ*_1_ (ns)	*A*_2_	*τ*_2_ (ns)	*τ*_avg_ (ns)
CdTe-pure	0.54	6.74	0.46	27.53	22.83
CdTe:Mn^2+^	0.21	14.61	0.79	55.73	53.27
CdTe:Co^2+^	0.64	14.52	0.36	50.34	38.10
CdTe:Mn^2+^&Co^2+^	0.30	17.25	0.70	64.67	59.83

As shown in Fig. 4, Mn^2+^ doping significantly improves the optical performance of CdTe QDs than Co^2+^ doping. However, when Co^2+^ and Mn^2+^ co-doped, the Co^2+^-doping energy level is located between the conduction bands of CdTe and Mn^2+^-doping energy level, which is conducive to the transition of electrons. This is essential to enhance the performance of QDSCs.

### 3.3. Properties of Mn^2+^ and Co^2+^ doped CdTe quantum dot for sensitized solar cells

**Fig. 5:**
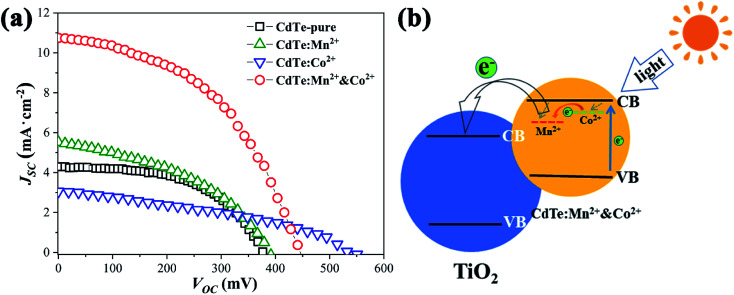
(a) *J*_SC_–*V*_OC_ curves for solar cells sensitized of CdTe-pure, CdTe:Mn^2+^, CdTe:Co^2+^ and CdTe:Mn^2+^&Co^2+^; (b) schematic representation of the energy diagram of CdTe:Mn^2+^&Co^2+^.

Fig. 5a shows *J*_SC_–*V*_OC_ curves of QDSCs sensitized by the samples prepared with various precursors. The data such as photocurrent (*J*_sc_), open circuit voltage (*V*_oc_), fill factor (FF) and efficiency (*η*) of corresponding cells are listed in Table 2, ten devices are tested, the statistical PCE present in Fig. S2,† the results show that the devices have good repeatability. Compared with sensitizer of CdTe-pure, the *J*_sc_ increases as doped Mn^2+^ (from 4.29 to 5.52, mA cm^−2^), due to doping Mn^2+^ can harvest more light range (see Fig. 4a) and the energy levels are matched electrons are more easily transferred from the Mn^2+^-doping energy level to the conduction band of TiO_2_. When CdTe QDs doped only Co^2+^, an increase of charge accumulation at the interface of QDs/TiO_2_ under illumination, therefore brings forward a greater upward shift of the TiO_2_ conduction band edge after sensitization and results in enhancement in photovoltage (from 384 to 550, mV) of the resultant cell devices.^27,31^ However, the *J*_sc_ decreases a lot (from 4.29 to 3.05, mA cm^−2^), the possible reason is that electrons and holes are easily recombined at the Co^2+^ doping level. For the sensitizer of CdTe:Mn^2+^&Co^2+^, both *V*_oc_ (453 mV) and *J*_sc_ (10.74 mA cm^−2^) are remarkably greater than those of pure CdTe QDs (384 mV and 4.29 mA cm^−2^). As analyzed in Fig. 4c, the doping energy level formed by Mn^2+^ is lower than that by Co^2+^. Therefore, the schematic diagram of the energy diagram of CdTe:Mn^2+^&Co^2+^ is given as shown in Fig. 5b. As the sunlight irradiated, the electrons are excited to the conduction band of the CdTe QDs. The excited electrons transit to the Co^2+^-doping and to the Mn^2+^-doping energy levels, then finally injected into the conduction band of TiO_2_, making the electrons avoid recombination at Co^2+^-doping level and easier to transfer into TiO_2_, resulting in the *J*_sc_ increase significantly. Meanwhile, doping Co^2+^ will still induce an increase of charge accumulation at the interface of QDs/TiO_2_ under illumination and therefore brings forward upward shift of the TiO_2_ conduction band edge after sensitization and results in enhancement in *V*_oc_ of the cell devices. Accordingly, Co^2+^ and Mn^2+^ co-doping can enhance the PCE of QDSCs. The efficiency is enhanced from 0.87% to 2.26%, which is more than 159% enhancement using a single doping procedure.

**Table 2:** Photovoltaic parameters of the QDSCs prepared by various sensitizers

Sensitizer	*τ* (ns)	*V*_oc_ (mV)	*J*_sc_ (mA)	FF	*η* (%)
CdTe-pure	22.83	384.00	4.29	0.53	0.87
CdTe:Mn^2+^	53.27	392.00	5.52	0.42	0.91
CdTe:Co^2+^	38.10	550.00	3.05	0.37	0.62
CdTe:Mn^2+^&Co^2+^	59.83	453.00	10.74	0.46	2.26

Doped QDs with various transition metals have a general beneficial effect on the performance of QDSCs. Recombination of electrons and holes is limited with doping of Mn^2+^ as the d–d transition is orbit ally forbidden,^35^ Co^2+^ doping make electrons transit to the conduction band of TiO_2_ easier, and also increases the *V*_oc_, which is benefit to the enhancement of the performance of QDSCs. In addition, red shifts in optical absorption of the sample induce an enhancement in optical absorption of AM 1.5G. Therefore, combine Mn^2+^ and Co^2+^ complement each other is a better way for the design of higher efficiency QDSCs.

## 4. Conclusions

Mn^2+^-, Co^2+^-doped CdTe QDs and Mn^2+^&Co^2+^ co-doped CdTe QDs with narrow size distribution were successfully synthesized *via* aqueous phase method with MPA. All of the samples possess zinc blende crystal structure. Doping Mn^2+^ and Co^2+^ is more conducive to improving optical properties compared with pure CdTe QDs due to the new doping energy levels. Combine Mn^2+^ and Co^2+^ complement each other, broader light harvesting range of the doped CdTe QDs has been observed. The retarded charge recombination have been confirmed by PL decay characterizations. As observed by PL decay measurements, doping with both Mn^2+^ and Co^2+^ result in a significant increase in the emission lifetime and high PLQY. In addition, QDSCs sensitized by the Mn^2+^&Co^2+^ co-doped QDs possesses much higher efficiency than that of sensitized by the pure CdTe QDs.

## Conflicts of interest

The authors declare no conflicts of interest in relation to this paper.

## Supplementary Material

RA-010-D0RA06381A-s001
