# Supplementary material for: The design of Mn2+&Co2+ co-doped CdTe quantum dot sensitized solar cells with much higher efficiency
Source: RSC Adv. 2020 Sep 29;10(59):35701–8. doi: 10.1039/d0ra06381a (PMC9056921; doi:10.1039/d0ra06381a)
Supplement: RA-010-D0RA06381A-s001 [file RA-010-D0RA06381A-s001.pdf]

Electronic Supplementary Material (ESI) for RSC Advances.
This journal is © The Royal Society of Chemistry 2020

Supporting Information

The design of Mn^{2+}&Co^{2+} co-doped CdTe quantum dots sensitized solar cells with much higher efficiency

Huazheng Li[a], Wangwei Lu[a], Bin Song[b], Jing Zhou[c], Gaoling Zhao[a*], Gaorong Han[a]

[a] State Key Laboratory of Silicon Materials & School of Materials Science and Engineering, Zhejiang University, Hangzhou 310027, P. R. China

[b] State Key Laboratory of Silicon Materials and Department of Physics, Zhejiang University, Hangzhou 310027, P. R. China.

[c] Department of Traffic Management Engineering, Zhejiang Police College, Hangzhou, 310053, P. R. China

*Corresponding author, E-mail: glzhao@zju.edu.cn.

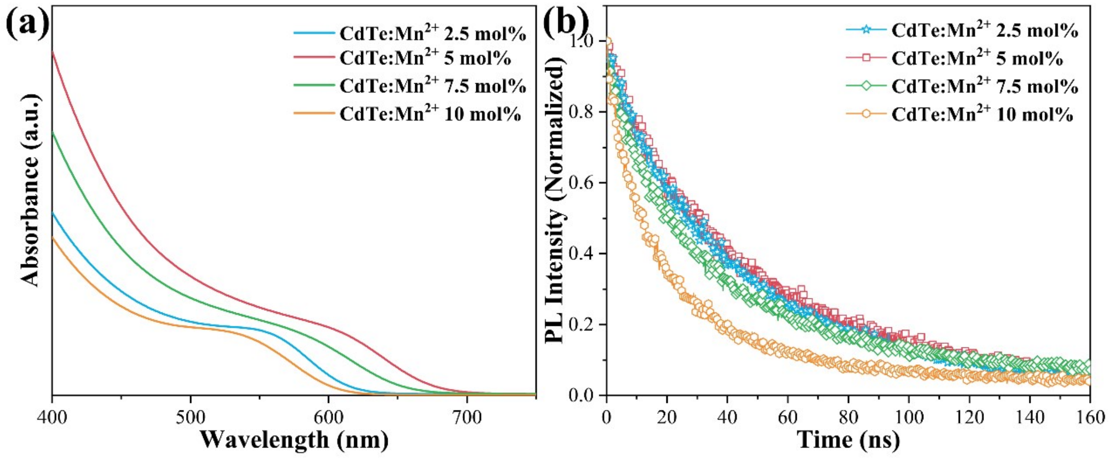

Figure S1. Optical absorption spectra (a) and PL decay spectra (b) of CdTe QDs prepared with various concentration of Mn precursor.

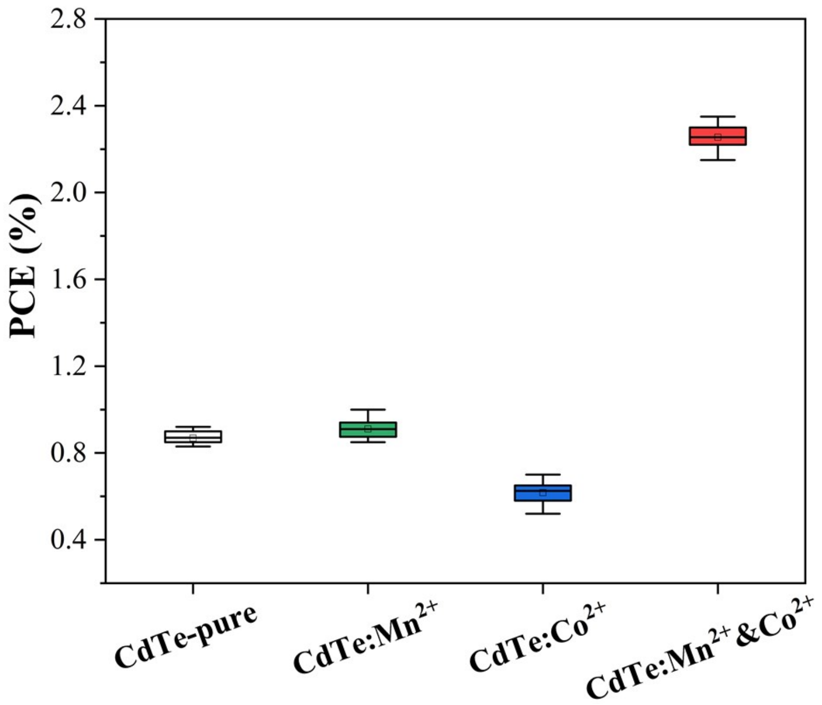

Figure S2. Statistical PCE of the fabricated QDSCs based on various sensitizers (CdTe-pure, CdTe:Mn^{2+}, CdTe:Co^{2+} and CdTe:Mn^{2+}&Co^{2+}).
